# Short‐Term Effects of Counselling Versus Occlusal Splint Therapy in TMD Pain Patients on Neck Pain and Dysfunction: A Randomised Trial

**DOI:** 10.1111/joor.70040

**Published:** 2025-09-04

**Authors:** Roberta Cimino, Rosaria Bucci, Roberto Rongo, Ambrosina Michelotti, Andrea Deregibus

**Affiliations:** ^1^ Division of Orthodontics and Temporomandibular Disorders, Department of Neurosciences, Reproductive Sciences and Oral Sciences, Postgraduate School of Orthodontics University of Naples Federico II Naples Italy; ^2^ Postgraduate School of Orthodontics and Gnathology Unit, Department of Surgical Sciences Dental School, University of Turin Turin Italy

**Keywords:** cervical pain, occlusal splints, temporomandibular joint disorders

## Abstract

**Background and Objective:**

Cervical spine impairments are frequent findings among patients with temporomandibular disorders (TMDs). Previous studies have demonstrated that treatments targeting the upper cervical region can improve pain symptoms in patients with TMD. The aim of the current study was to assess whether counselling alone and counselling plus occlusal splint therapy can also provide relief for coexisting neck pain.

**Subjects and Methods:**

Consecutive adult patients with TMD pain and neck dysfunction were randomised into one treatment group (counselling only—C, or counselling plus occlusal splint—OS). Neck pain was measured using a VAS scale, while neck disability was computed using the Neck Disability Index (NDI). Treatment outcomes were measured after 3‐month follow‐up.

**Results:**

Twenty‐seven patients were recruited for the C group (5 males, 22 females, mean age ± SD: 36.5 ± 11.8 years), while 24 belonged to the OS group (3 males, 21 females, mean age ± SD: 35.5 ± 12.5 years). After the interventions, neck pain score and NDI were significantly reduced in both groups, and no between‐groups difference was observed.

**Conclusion:**

Both counselling alone and along with occlusal splint therapy effectively reduce neck pain and improve neck function in TMD patients. The findings suggest that occlusal splint does not provide any additional value, as compared to counselling alone, in the achievement of the treatment outcomes.

## Graphical abstract

In the comparison between counselling only and counselling + occlusal splint therapy, no differences where found in terms of reduction of neck pain and neck dysfunction.

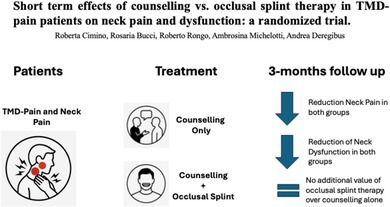

**Abbreviations**
C: counselling
NDI: neck disability index
OS: occlusal splint
TMD: temporomandibular disorders
TMJ: temporomandibular joint

## 1 Introduction

Temporomandibular disorders (TMDs) are a group of musculoskeletal conditions that affect the temporomandibular joints (TMJs), the masticatory muscles and associated tissues. Symptoms of TMD typically include pain (in the face, mouth or jaw) and/or dysfunction, such as joint noises or restricted jaw movement. Most commonly, patients report that their symptoms are influenced by mandibular activity, including eating, chewing and speaking [1, 2]. TMDs are among the most frequent causes of chronic orofacial pain and can significantly impact a patient's quality of life. Their aetiology is multifactorial, involving a combination of biomechanical, neuromuscular and psychosocial factors [3]. Many patients with TMD also suffer from other comorbidities, such as headache, neck and back pain, chronic fatigue syndrome, fibromyalgia and irritable bowel syndrome [4, 5, 6].

The connection between TMD and neck pain has gained attention in scientific literature. Research has shown that individuals with TMD often report co‐occurring neck pain and stiffness, with some studies suggesting that up to 70% of TMD patients experience some form of cervical discomfort [7, 8, 9]. This relationship is likely due to the biomechanical and neuromuscular interplay between the TMJ and cervical spine. The TMJ and cervical spine share common muscle groups, including the sternocleidomastoid and trapezius, which can contribute to the spread of pain. Furthermore, the close anatomical proximity and functional integration between the jaw and neck mean that dysfunction in one area may influence the other [10].

This intricate connection has led to the observation of decreased TMD pain symptoms after treatment aimed at the upper cervical spine region [11, 12, 13], thus supporting the importance of looking at cervical spine function when evaluating subjects with TMD. Similarly, it has been hypothesised that dealing with TMD symptoms may have a positive impact on cervical spine‐related issues, including neck pain and dysfunction.

Recent international consensus underlined that TMD treatment should primarily be based on encouraging supported self‐management and conservative approaches [14, 15]. Counselling is a conservative and low‐cost approach that carries no risks, making it an attractive first‐line treatment for TMD‐related symptoms [16], including coexisting neck pain. Occlusal splint therapy is also a widely used non‐invasive treatment modality for TMD that could potentially offer benefits that extend beyond the immediate orofacial region [2, 17]. Although the actual mechanism of action is still unclear, these splints are thought to work by redistributing occlusal forces, reducing excessive muscular activity and promoting muscle relaxation, all of which contribute to the reduction of TMD‐related pain and joint loading [18].

Numerous studies have documented the efficacy of occlusal splint therapy in alleviating common symptoms of TMD, such as jaw pain, joint sounds and headaches. However, the potential effects of splints on secondary symptoms, particularly those related to the cervical spine, have received comparatively less attention. Given the overlap between TMD and neck pain, investigating whether occlusal splint therapy can positively impact neck pain and dysfunction is crucial for providing a more comprehensive treatment strategy for TMD patients. Most clinical studies have focused on the efficacy of occlusal splints in reducing jaw pain and improving TMJ function, with few studies addressing the broader musculoskeletal outcomes, such as neck‐related symptoms [19].

Given that counselling (self‐management education) is a proven, low‐cost intervention for TMD, it is clinically important to determine whether adding an occlusal splint provides any additional benefit for neck pain and dysfunction. Therefore, the present randomised clinical trial aims to investigate the short‐term effects of occlusal splint therapy on neck pain and dysfunction in patients with TMD‐related pain, and to compare it with the effect of self‐management alone. The primary objective was to assess whether therapies commonly used to manage TMD can also provide relief for coexisting neck pain. Secondary objectives include evaluating the effects of TMD therapies on neck‐related functional outcomes.

We hypothesise that occlusal splint therapy, by addressing the abnormal forces and muscular hyperactivity associated with TMD, could result in a significant reduction in both TMD‐related pain and neck pain, and that this reduction could be superior to that obtained with counselling alone. Furthermore, we expect to observe improvements in neck function, as measured by subjective reports of disability. The results of this study will contribute to a growing body of literature on the interconnectedness of the TMJ and cervical spine and may provide clinicians with a more holistic understanding of the benefits of TMD therapy for patients with TMD and neck pain.

## 2 Materials and Methods

For the purpose of this perspective randomised clinical trial, all patients signed a written informed consent. The study was approved by the Ethics Committee of University of Turin (approval no. 246/2019).

### 2.1 Participants

Patients were consecutively recruited from those seeking treatment between March 2019 and December 2019 at the Clinic of TMD of the CIR Dental School of University of Turin (Turin, Italy). The inclusion criteria were: adult patients (18–60 years of age); pain‐related TMD diagnosis (myalgia and/or arthralgia and/or headache attributed to TMD) according to Diagnostic Criteria for TMD [2]; current level of TMD pain > 3 assessed on a numerical rating scale (Question 2 of the ‘Graded Chronic Pain Scale’ [20]); presence of current neck pain > 3 assessed on a numerical rating scale. The TMD examination was performed by one calibrated operator (RC). The exclusion criteria were: other therapy for TMD pain or cervical spine pain in the preceding 3 months; pharmacological therapy (NSAID, muscle‐relaxing drugs, tricyclic antidepressant) in the last month; systemic neurologic diseases; previous facial trauma, whiplash or TMJ surgery.

Patients were randomly assigned using a predetermined block randomisation list prepared by an independent statistician. The allocation was concealed until the point of assignment, when the investigator (RC) enrolled each patient into the next sequential group per the randomisation sequence. Owing to the nature of the treatments, neither participants nor the treating clinician could be blinded. However, the statistician performing the data analysis was kept blinded to group allocations. One group received counselling plus occlusal splint therapy (group OS), while the other group received counselling only (group C). Patients were considered lost during follow‐up whenever they did not attended the clinical appointment scheduled after 3 months of treatment.

### 2.2 Treatment Modalities

COUNSELLING—Counselling included a patient education programme, comprising general information about self‐care of jaw musculature, already used in previous studies [17]. One operator (RC) reassured the patient by explaining the benign nature of TMD, the possible aetiology, and the good prognosis of these disorders. Furthermore, explanations about the normal jaw muscles function and the possible overuse of these muscles as a cause of their complaints were provided. The patients were instructed to carefully and constantly control their muscle activity, to avoid oral parafunctional habits or excessive mandibular movements and to keep a soft diet. Patients were also instructed to keep their jaw muscles relaxed, by holding the mandible in a rest position with teeth apart. The mandibular rest position was determined by pronouncing the letter ‘N’ several times and keeping the tongue behind the upper incisor teeth, with the lips in slight contact. Finally, patients were invited to keep practising what they learned at home and during their common activities, by using audio/visual reminders.

OCCLUSAL SPLINT (OS)—Alginate impressions were taken to fabricate hard resin stabilisation (Michigan type) upper OS. The characteristics of the OS were: flat surface, full coverage of occlusal upper dental surfaces (or lower, when patients presented reduced dental support), uniform contact points, short coverage of the labial surfaces and buccal surfaces of the maxillary teeth to provide frictional retention. Patients were instructed to wear the occlusal splint every night and to follow the counselling instructions (exercises and habit modifications) daily; however, no formal measure of adherence (e.g., usage diaries or compliance checks) was employed during the 3‐month follow‐up.

### 2.3 Timepoint and Outcomes

Study outcomes were collected at the baseline, when patients received written instructions about their own treatment programme (T0) and after 3 months (T1). The operator instructed the patients to continue with the prescribed therapy throughout a three‐month period even if they were pain free. During the entire study period, no participants received any other form of treatment other than that assigned to their group.

Study outcomes were TMD pain, neck pain, neck disability, jaw opening. TMD pain and neck pain were measure as maximum spontaneous pain during the last 30 days, using a VAS (Visual Analogue Scale) made of a horizontal segment of 100 mm, whose anchors corresponded to ‘No Pain’ (left anchor) and ‘Worst Pain Imaginable’ (right anchor). Neck disability was recorded using the Italian version of the Neck Disability Index (NDI) questionnaire [21, 22]. The NDI is a 10 questions questionnaire, with each item being possibly rated on a 0 to 5 rating scale. According to the sum of all item (maximum score 50 points), the disability could be interpreted as ‘none’ (from 0 to 4), ‘mild’ (from 5 to 14), ‘moderate’ (from 15 to 24), ‘severe’ (from 25 to 34) and ‘complete’ (higher than 34). Jaw opening was measured as the maximal distance (in mm) between the maxillary and mandibular incisal edges during a pain free mandibular opening movement.

### 2.4 Statistical Analysis

A total sample size of 64 patients (32 for each group) achieves 80% power to detect a difference of 10.0 under the null hypothesis that group means are equal, considering group standard deviations of 14.0 and a significant level (alpha) of 0.05 using a two‐sided two‐sample means test. The power analysis was calculated on NDI values.

Statistician was blinded to the group assignment. All randomised patients were included in the summary statistics. The basic characteristics of the patients were reported as absolute and relative frequencies for categorical variables and as means and standard deviations or medians and interquartile ranges for continuous variables. For each parameter collected, the main descriptive statistics were provided (means and standard deviations for continuous data). Statistical analyses were performed on a per‐protocol basis, including only participants who completed the 3‐month follow‐up. Participants who were lost to follow‐up had no post‐treatment data and thus could not be included in an intention‐to‐treat analysis. No data imputation was performed for dropouts.

Differences between the two groups at time T1 were evaluated using the chi‐square test or Fisher's exact test for categorical variables, and the Mann–Whitney *U*‐test or *t*‐test for continuous variables. The choice between parametric and non‐parametric tests was determined based on the results of the Shapiro–Wilk normality test. Confidence intervals were calculated at 95%, and the significance level was set at 5%. The NDI and VAS indices were analysed both as continuous variables and as categorical variables (using clinically representative categories). Effect sizes were calculated to quantify the magnitude of differences between groups. Cohen's *d* was computed using standard formulas; for asymmetric variables, an approximate method was applied to estimate the standardised mean difference. All statistical analyses were performed using R software.

## 3 Results

**FIGURE 1:**
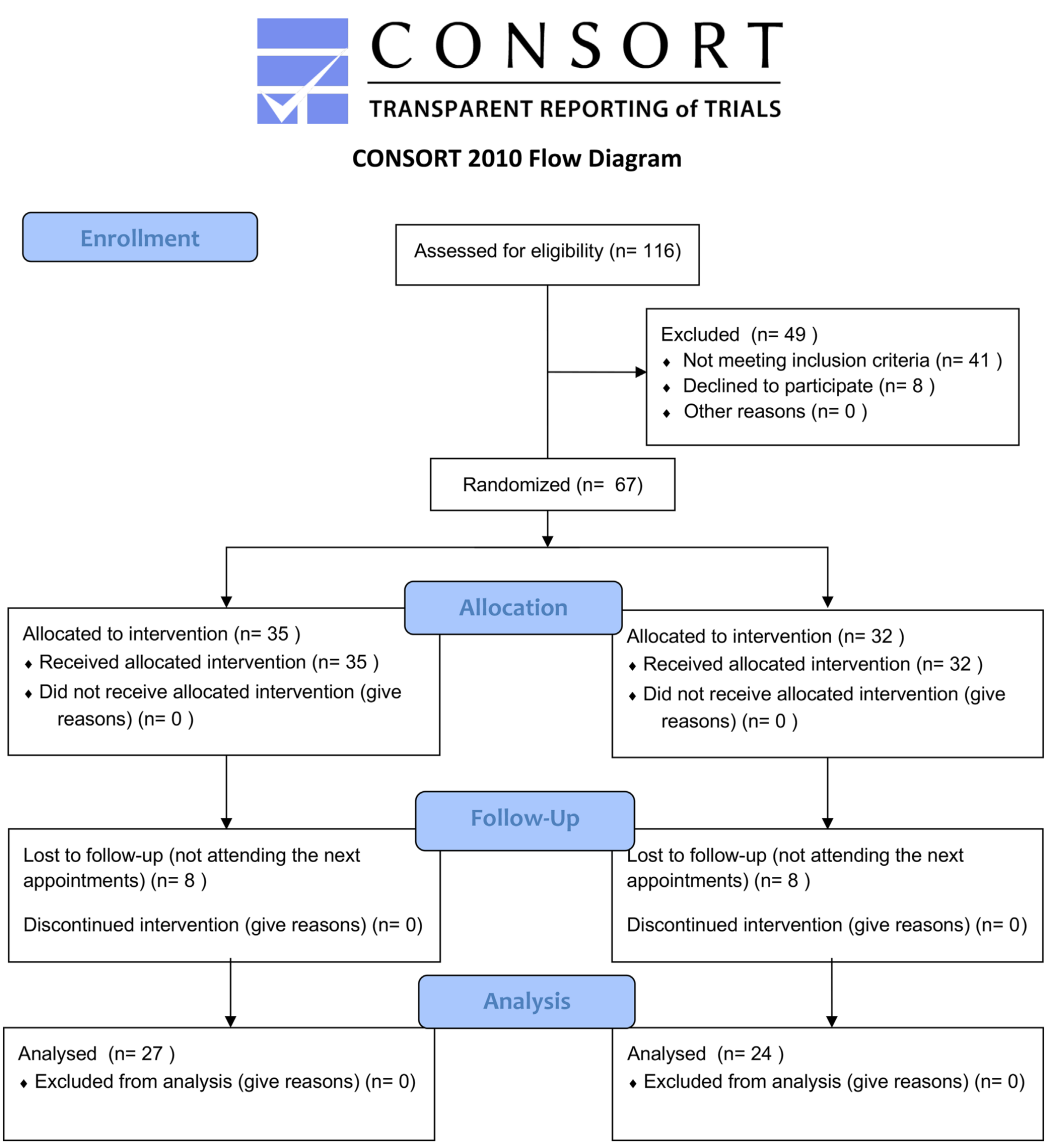
CONSORT 2010 flow diagram.

During the recruitment period, a total of 116 patients were assessed for eligibility. Of these, 49 were excluded (due to not meeting inclusion criteria or declining participation) and 67 eligible patients were enrolled and randomised into two groups. Sixty‐seven (67) patients were recruited and allocated in the two study groups: 35 patients received counselling only, while 32 patients received counselling and occlusal splint. In each group, 8 patients were lost during follow‐up due to several reasons (moved to other cities, refused to attend the next appointment, did not show up). The final sample size consisted of 52 patients (Figure 1), of which 27 belonged to the C group (5 males, 22 females, mean age ± SD: 36.5 ± 11.8 years), while 24 belonged to the OS group (3 males, 21 females, mean age ± SD: 35.5 ± 12.5 years).

The TMD diagnoses of the included patients are reported in Table 1. In both groups, the majority of the participants were diagnosed with myalgia and/or headache attributed to TMD, while arthralgia was less prevalent. No significant difference was found concerning the TMD diagnosis in the two groups (all *p* > 0.05).

**TABLE 1:** Distribution of the TMD diagnosis, according to the DC/TMD in the two study groups (C, counselling; OS, occlusal splint therapy).

	Group C	Group OS
	*n* = 27	*n* = 24
Myalgia, *n* (%)	27 (100%)	23 (96%)
Arthralgia, *n* (%)	12 (44%)	9 (38%)
Headache attributed to TMD, *n* (%)	23 (85%)	22 (92%)

Regarding the pain‐free Maximum Mouth Opening (MMO), a significant increase (2.6 ± 5.8 mm) was observed comparing the 3‐month follow‐up with the baseline values of the C group (*p* = 0.03), while no significant difference was observed in the OS group. No difference was also found in the between‐group comparison of the delta scores (*p* = 0.64, Table 2).

**TABLE 2:** Pre‐treatment (T0), post‐treatment (T1) values and differences (T1–T0) for each study variable in the two study groups (C, counselling; OS, occlusal splint therapy).

		T0			T1			Diff. T1–T0			Effect size
											C versus OS at T1
		Group C	Group OS	*p*	Group C	Group OS	*p*	Group C	Group OS	*p*	Cohen's *d* (95% CI)
				C versus OS			C versus OS			C versus OS	
MMO (mm)	Mean (SD)	41.6 (9.3)	43.5 (7.2)	0.430	44.2 (6.7)	45.1 (6.9)	0.640	2.6 (5.8)	1.8 (5.7)	0.640*	0.14 (−0.41, 0.69)
Neck‐P (mm)	Median (IQR)	76.0 (63.0, 85.0)	73.0 (45.5, 83.5)	0.310	64.0 (48.0, 76.0)	65.0 (41.0, 74.0)	0.760	−7.0 (−23.0, 3.0)	−8.0 (−14.0, −1.0)	0.690^†^	−0.12 (−0.67, 0.42)
TMD‐P (mm)	Mean (SD)	69.4 (21.2)	56.9 (21.7)	**0.043**	61.4 (25.5)	49.2 (27.3)	0.110	−8.0 (30.4)	−9.4 (27.2)	0.860*	0.05 (−0.5, 0.6)
NDI	Mean (SD)	13.0 (5.3)	11.3 (5.1)	0.240	9.8 (6.2)	7.4 (5.1)	0.150	−3.3 (5.9)	−4.2 (5.3)	0.570*	0.16 (−0.39, 0.71)

*Note:* Statistically significant differences are reported in bold.

Abbreviations: MMO, maximum mouth opening; NDI, neck disability index; Neck‐P, neck pain; TMD‐P, temporomandibular disorder pain.

* Paired‐sample *t*‐test.

^†^ Wilcoxon's rank‐sum test.

VAS score for neck pain was significantly reduced after treatment in both groups (Group C: −7.0 mm, *p* = 0.006; Group OS: −8.0 mm, *p* = 0.02), but no differences were found between the study groups (*p* = 0.69, Table 2).

At baseline, the counselling‐only group reported higher TMD pain levels (mean VAS) than the occlusal splint group; this difference was statistically significant (*p* < 0.05). Although both groups showed a trend of pain reduction over time, the baseline imbalance necessitates caution when directly comparing the magnitude of pain improvements between groups (Table 2).

Neck disability, as measured with the NDI, was reduced in both groups across time (Group C: −3.3, *p* = 0.007; Group OS: −4.2, *p* = 0.001). This improvement was not significantly different between the two study groups (*p* = 0.57, Table 2).

The estimated effect size was small and not clinically meaningful (Cohen's *d* < 0.5); supporting the main analysis and confirming the absence of relevant differences between the groups (Table 2).

## 4 Discussion

The current randomised clinical trial aimed to investigate the effects of counselling plus night‐time use of an occlusal splint, which is commonly used for TMD pain management, on neck pain and neck disability. The comparator was a group of TMD pain patients undergoing counselling therapy only. In both groups, neck pain and self‐report of disability were significantly reduced after 3 months of treatments.

A follow‐up at 3 months was chosen to assess short‐term outcomes, since prior TMD intervention studies have demonstrated that significant changes in pain and disability can be detected within this timeframe. This duration provides an initial measure of efficacy while limiting attrition. The TMD diagnosis data revealed that most participants in both groups were diagnosed with myalgia and headache attributed to TMD, while arthralgia was less prevalent. Importantly, no significant difference in TMD diagnosis between the counselling and occlusal splint therapy groups was found, ensuring that the baseline characteristics of the participants were well matched. This homogeneity in diagnosis strengthens the comparability of the two intervention groups and supports the validity of the study's results.

The primary functional outcome, pain‐free Maximum Mouth Opening (MMO), showed a significant improvement in the counselling group, with an increase of 2.6 ± 5.8 mm over the three‐month follow‐up period. In contrast, the occlusal splint group did not experience a statistically significant change in MMO, even though counselling was prescribed also in this group of patients. These results suggest that counselling alone, which may involve self‐massages and exercises, could be effective in improving jaw mobility over a short time period, and this improvement may be attributed to the focus on patient education and behavioural modifications aimed at reducing muscle tension and encouraging normal jaw function. However, patients who received the occlusal splint alongside instruction for counselling therapy might have reduced their attention on self‐management, relying more on the effect of the device, and thus showing less improvement in the MMO not reaching statistical significance.

Both groups demonstrated significant reductions in neck pain, as measured by the VAS score, after treatment, indicating that both interventions are effective in reducing neck pain in TMD patients. Importantly, no significant between‐group differences were observed, suggesting that the two treatment modalities may provide comparable benefits in terms of neck pain relief. One possible explanation for the equivalent outcomes is that both interventions ultimately act on the same pathways of pain reduction. Counselling addresses muscle tension and pain through stress reduction and exercises (behavioural mechanisms), while the occlusal splint acts by biomechanically stabilising the jaw and changing masticatory muscle activity. Both approaches may reduce cervical muscle strain and pain, so the combination might not yield a supra‐additive effect. Additionally, patients in the splint group may have inadvertently placed less emphasis on their self‐management routines (given the presence of a device), which could limit the incremental benefit of the splint. These factors could explain why no additional improvement was observed with occlusal splint use in this short‐term period. This aligns with previous studies that have highlighted the biomechanical relationship between the TMJ and cervical spine, wherein addressing TMD symptoms can positively influence cervical discomfort [23, 24]. Several theories have been proposed to explain how occlusal splints might influence neck pain. One hypothesis is that by reducing excessive or abnormal loading on the TMJ and the associated muscles of mastication, splints may indirectly decrease muscular tension in the neck region, leading to a reduction in pain and improved function. Another theory is that occlusal splints could normalise the posture of the jaw and cervical spine, thereby reducing the biomechanical strain on the neck muscles [25]. Additionally, the use of occlusal splints may decrease peripheral and central sensitisation, a phenomenon where the nervous system becomes overly responsive to stimuli, contributing to widespread pain, including in the neck [26]. Although neck pain scores significantly decreased in both groups, the mean reduction (~7%–8%) was relatively small. In chronic pain terms, a change of this magnitude may be below the typical threshold for a clinically noticeable improvement. Similarly, the NDI scores improved by 3–4 points, which, while statistically significant, represents only a mild improvement in disability (remaining within the ‘mild disability’ category).

In contrast, the VAS score for TMD‐related pain revealed a baseline difference between the groups, with the counselling group reporting significantly higher levels of pain compared to the occlusal splint group. Although a trend toward pain reduction was observed in both groups over time, no significant differences were found across times or between groups. This imbalance could affect the interpretation of the results: a group starting with more pain has more room to improve, but also their final pain levels remain difficult to compare with a group that started lower. This could have obscured subtle between‐group differences. Given this as a limitation, these findings suggest that both counselling and occlusal splint therapy can potentially alleviate TMD pain, but the short‐term effects may be subtle and require a longer follow‐up to detect significant changes. Additionally, the baseline difference in TMD pain scores could have influenced the ability to detect more substantial between‐group differences in pain reduction.

Neck disability, as measured by the Neck Disability Index (NDI), improved significantly in both groups. The occlusal splint group experienced a reduction of −4.2 points, while the counselling group saw a reduction of −3.3 points. These improvements reflect meaningful reductions in neck‐related dysfunction, further supporting the effectiveness of both treatment modalities in addressing the functional aspects of neck pain in TMD patients. Similar to the VAS for neck pain, no significant differences were found between the two groups, suggesting that occlusal splints and counselling are similarly effective in reducing neck disability.

The improvement in neck function observed in both groups can be attributed to the shared neuromuscular pathways between the TMJ and the cervical spine. Previous research has demonstrated that interventions targeting neck pain can have a positive impact on TMD [12, 27]. The counselling group may have benefited from behavioural modifications aimed at reducing stress and muscle tension, while the occlusal splint group may have experienced improvements through the stabilisation and redistribution of occlusal forces.

The comparable effects of counselling and occlusal splint therapy on neck pain and dysfunction have important clinical implications for the management of TMD patients. The comparable effects of counselling and counselling plus splint therapy indicate that counselling alone—a low‐cost and non‐invasive approach—may be sufficient as an initial treatment for TMD patients with concurrent neck pain. In practice, this means clinicians can confidently implement patient education and self‐management strategies as a first‐line therapy, reserving occlusal splint therapy for cases where additional benefit is needed. This supports a conservative management strategy and aligns with the view that addressing behavioural and lifestyle factors (through counselling) can significantly impact musculoskeletal pain [14, 15, 28].

While the study provides valuable insights, several limitations should be acknowledged. First, the baseline difference in VAS for TMD pain between groups could have influenced our ability to detect between‐group differences in pain reduction. This imbalance might have provided an initial advantage to the occlusal splint group and thus biased the comparative outcomes. Future studies should consider stratified randomisation or adjust for baseline pain to mitigate this issue. Secondly, the study's power was reduced by the dropouts, and the absence of an ITT analysis is acknowledged as a limitation, as it could affect the robustness of our findings. Also, it is recognised that a 3‐month follow‐up is a relatively short period, and prolonged follow‐up would be necessary to confirm the long‐term sustainability of the treatment effects. However, the fluctuating nature of TMDs might influence the long‐term results. Finally, the self‐reported nature of some outcomes, such as the VAS for pain, may be subject to recall bias or individual variability in pain perception.

## 5 Conclusions

In conclusion, both occlusal splint therapy and counselling effectively reduce neck pain and improve neck function in TMD patients; no additional benefit was detected from occlusal splint therapy compared to counselling alone. Although the occlusal splint group experienced a baseline advantage in TMD‐related pain, both groups showed a trend toward pain reduction over time. These findings suggest that clinicians can consider both approaches for managing neck pain and dysfunction in TMD patients, with counselling offering a less invasive alternative.

## Author Contributions

Conceptualization: R.C. and A.M.; methodology: R.C., A.M. and A.D.; formal analysis: R.B. and R.R.; investigation: R.C.; resources: A.D.; data curation: R.B.; writing – original draft preparation: R.B.; writing – review and editing: R.C. and R.R.; visualization: R.R.; supervision: R.C., A.M. and A.D. All authors have read and agreed to the published version of the manuscript.

## Disclosure

Disclaimer: The statements, opinions and data contained in all publications are solely those of the individual author(s) and contributor(s) and not of MDPI and/or the editor(s). MDPI and/or the editor(s) disclaim responsibility for any injury to people or property resulting from any ideas, methods, instructions or products referred to in the content.

## Ethics Statement

The study was conducted in accordance with the Declaration of Helsinki, and approved by the Ethics Committee of the University of Turin (no. 246/2019).

## Consent

Informed consent was obtained from all subjects involved in the study.

## Conflicts of Interest

The authors declare no conflicts of interest.

## Data Availability

The raw data supporting the conclusions of this article will be made available by the authors on request.
